# Small compounds mimicking the adhesion molecule L1 improve recovery in a zebrafish demyelination model

**DOI:** 10.1038/s41598-021-85412-1

**Published:** 2021-03-15

**Authors:** Suhyun Kim, Dong-Won Lee, Melitta Schachner, Hae-Chul Park

**Affiliations:** 1 Department of Biomedical Sciences, College of Medicine, Korea University, Ansan, 15335 Republic of Korea; 2 Keck Center for Collaborative Neuroscience and Department of Cell Biology and Neuroscience, Rutgers University, Piscataway, NJ 08554 USA; 3 Center for Neuroscience, Shantou University Medical College, Shantou, 515041 Guangdong China

**Keywords:** Myelin biology and repair, Phenotypic screening

## Abstract

Demyelination leads to a loss of neurons, which results in, among other consequences, a severe reduction in locomotor function, and underlies several diseases in humans including multiple sclerosis and polyneuropathies. Considerable clinical progress has been made in counteracting demyelination. However, there remains a need for novel methods that reduce demyelination while concomitantly achieving remyelination, thus complementing the currently available tools to ameliorate demyelinating diseases. In this study, we used an established zebrafish demyelination model to test selected compounds, following a screening in cell culture experiments and in a mouse model of spinal cord injury that was aimed at identifying beneficial functions of the neural cell adhesion molecule L1. In comparison to mammalian nervous system disease models, the zebrafish allows testing of potentially promotive compounds more easily than what is possible in mammals. We found that our selected compounds tacrine and duloxetine significantly improved remyelination in the peripheral and central nervous system of transgenic zebrafish following pharmacologically induced demyelination. Given that both molecules are known to positively affect functions other than those related to L1 and in other disease contexts, we propose that their combined beneficial function raises hope for the use of these compounds in clinical settings.

## Introduction

Myelin is a multilayered sheath wrapped around axons that is important for rapid action potential conduction, survival, and metabolic support^1,2^. Various factors of genetic, immunological, and environmental origins can disrupt myelin structures and impair normal signal transduction, ultimately resulting in neurodegeneration^3^. Importantly, myelin defects, including demyelination, can be restored by oligodendrocytes. However, persistent demyelination and/or failure of remyelination can lead to several neurological disorders, such as multiple sclerosis (MS), leukomalacia, and Charcot Marie Tooth disease^4^. MS is a chronic demyelinating disease of the central nervous system, presently affecting approximately 2.5 million people worldwide. It is thought to be caused by the immune system inappropriately attacking myelin in the brain and spinal cord, resulting in the demyelination of axons^5,6^. Therapy for MS is often relatively successful via treatment with immunomodulators, such as interferon beta or a nuclear factor κB (NF-κB) inhibitor, although the outcome is a delay in disease progression, not regeneration. Even though therapy that combines the promotion of immunomodulation and remyelination has shown promise in animal models, there are only a few drugs that promote remyelination^7^. Among these, the FDA-approved drug benztropine, an inhibitor of acetylcholinesterase and antagonist of muscarinic M1 and M3 receptors, has been shown to promote oligodendrocyte precursor cell differentiation and remyelination^8^. Another compound, 4-aminopyridine, acts as a potassium channel inhibitor that exerts beneficial effects in patients with MS by restoring action potential activities in demyelinated axons^9^. Despite this progress in the treatment of MS, an urgent need remains for the identification of novel drugs to enhance remyelination, in combination with a modulation of the immune response.

The zebrafish is a useful model to study myelination in vivo based on several advantages, including genomic conservation, the availability of well-developed genetic tools, and transparency of the embryos. On account of evolutionary conservation from jawed fish to mammals, the molecular composition of myelin, its structure and gene expression profiles are highly similar^10^. Furthermore, the life cycle of zebrafish, which enables a relatively straightforward maintenance in the laboratory, is favorable for the screening and testing of bioactive compounds. To generate a zebrafish model useful for demyelination research, we developed *Tg(mbpa:gal4-vp16;uas:NTR-mCherry)* transgenic zebrafish, in which mature oligodendrocytes are conditionally ablated under the control of the *mbpa* promoter^11^. Expression of nitroreductase (NTR), which is encoded by the bacterial cytotoxin gene *nsfB*, is not cytotoxic; however, upon treatment with the prodrug metronidazole (MTZ), this enzyme converts MTZ into cytotoxins, thus enabling targeted cell ablation of oligodendrocytes and Schwann cells. Therefore, exposure of our transgenic zebrafish to an MTZ-containing medium results in rapid ablation of oligodendrocytes and Schwann cells, followed by subsequent demyelination within the nervous system.

L1 cell adhesion molecule (L1CAM; hereafter abbreviated L1) is a cell adhesion molecule with multiple functions in nervous system development, synaptic plasticity, and regeneration after injury^12^. It has been shown to exert beneficial effects in ameliorating the consequences of spinal cord injury and of Huntington’s, Parkinson’s, and Alzheimer’s diseases in animal models. However, the use of L1 in the treatment of these diseases is hampered by the large size of this protein, which is difficult to synthesize recombinantly or to isolate from tissues in larger quantities. To circumvent this issue, small organic compounds were isolated from an NIH library and found to elicit L1 functions by binding to the active site of L1 in the N-terminal part of the third fibronectin type III homologous repeat^12,13^. These compounds yielded beneficial effects in mouse and zebrafish spinal cord injury^14–16^. Out of these L1-mimicking compounds, we chose duloxetine and tacrine to investigate their effects in a zebrafish model of demyelination. It should be pointed out that duloxetine is a therapeutic drug for mood disorders and neuropathic pain, acting as a selective serotonin and norepinephrine reuptake inhibitor^17^. Tacrine is known to be a reversible acetylcholinesterase inhibitor^18^. Since L1 has been shown to contribute to myelination, we deemed it important to investigate whether these mimetic compounds are suitable for enhancing nervous system remyelination.

Here, we used the NTR-MTZ model zebrafish to investigate whether agonistic L1 mimetics would influence remyelination after experimentally induced demyelination. Our findings demonstrate that application of tacrine and duloxetine improved oligodendrocyte survival and enhanced remyelination, both in the central and peripheral nervous system. In addition, tacrine, but interestingly not duloxetine, restored the locomotive functions of demyelinated zebrafish larvae, which is in agreement with the observed neuroprotection at the cellular level.

## Results

### Tacrine and duloxetine promote the regeneration of oligodendrocytes in the central nervous system after oligodendrocyte ablation

**Figure 1:**
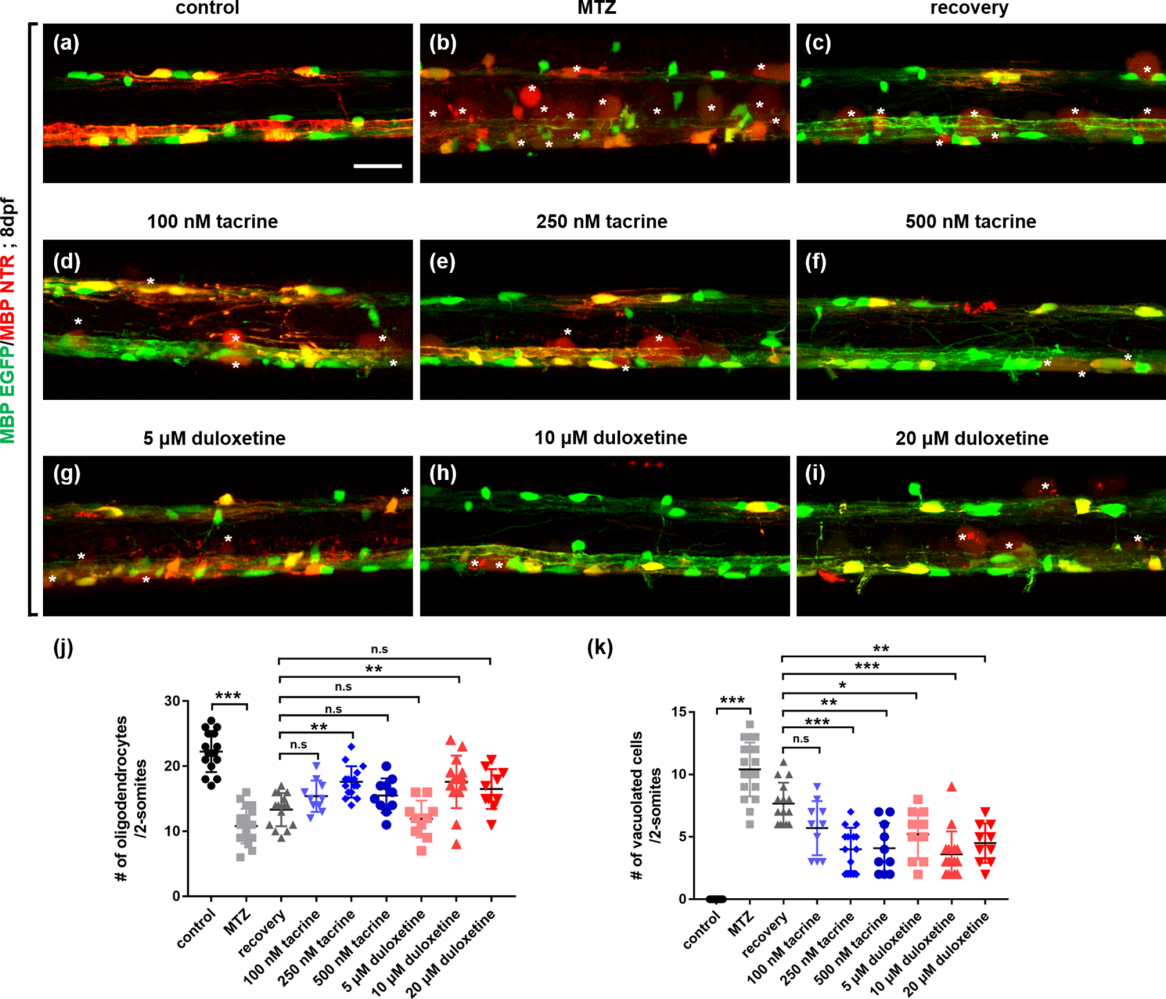
Effects of L1 mimetic compounds on the regeneration of oligodendrocytes in the spinal cord after oligodendrocyte ablation. All images are lateral views of the spinal cord of *Tg(mbpa:gal4-vp16;uas:gfp;uas:NTR-mCherry)* larvae at 8 days post-fertilization (dpf), anterior to the left and dorsal to the top. Fluorescence indicates MBP-positive oligodendrocytes and myelin sheaths in the spinal cord (**a–i**). White asterisks indicate debris of vacuolated oligodendrocytes. (**j**) Quantification of the number of oligodendrocytes per 2-somite area. Control: 22.27 ± 3.15, MTZ: 10.8 ± 2.90, recovery: 13.33 ± 2.55, 100 nM tacrine: 15.4 ± 2.41, 250 nM tacrine: 17.6 ± 2.41, 500 nM tacrine: 15.5 ± 1.64, 5 μM duloxetine: 11.9 ± 2.84, 10 μM duloxetine: 17.6 ± 4.05, 20 μM duloxetine: 16.5 ± 3.06. (**k**) Quantification of the number of vacuolated cells per 2-somite area. Control: 0, MTZ: 10.4 ± 2.36, recovery: 7.67 ± 1.68, 100 nM tacrine: 5.7 ± 2.16, 250 nM tacrine: 4 ± 1.73, 500 nM tacrine: 4.1 ± 2.02, 5 μM duloxetine: 5.2 ± 1.99, 10 μM duloxetine: 3.6 ± 1.84, 20 μM duloxetine: 4.5 ± 1.58. n = 15 for the control, recovery, 250-nM tacrine, and 10-μM duloxetine groups; n = 18 for the MTZ group; n = 10 for the 100- and 500-nM tacrine and 1-, and 20-μM duloxetine groups. ***p < 0.001; **p < 0.01; *p < 0.05; n.s., not significant. Scale bar, 25 μm.

To examine the effect of the two selected compounds on remyelination, we took advantage of the transgenic zebrafish model of demyelination using the NTR-MTZ system. *Tg (mbpa:gal4-vp16;uas:NTR-mCherry*) larvae express mCherry in oligodendrocytes (Fig. 1a and Supplementary Fig. 1). To induce oligodendrocyte ablation, 10 mM MTZ was used on larvae for 3 days, from days post-fertilization (dpf) 5–8 (Supplementary Fig. 2). Following MTZ treatment, larvae robustly showed vacuolated cells with fuzzy red fluorescence, cell death, and reduced myelin sheaths in the spinal cord (Fig. 1b). The number of oligodendrocytes was significantly lower in MTZ-treated larvae than in control transgenic larvae, which were left untreated. Following MTZ treatment for 2 days, larvae were washed to remove MTZ and incubated for another day in an embryonic medium containing 1% DMSO (hereafter referred to as the recovery group) or L1 mimetic compounds thereafter. The number of GFP-expressing oligodendrocytes in compound-treated larvae was determined in comparison to the recovery group, which showed a slight, non-significant restoration of oligodendrocyte numbers (Fig. 1c). Tacrine hydrochloride (hereafter referred to as tacrine) was applied at concentrations of 100, 250, and 500 nM (Fig. 1d–f). The group treated with 250 nM tacrine showed significantly improved oligodendrocyte regeneration compared to the MTZ-treated group and the recovery group (Fig. 1j). Additionally, the number of vacuolated cells was significantly reduced by tacrine at this concentration (Fig. 1e,k). (S)-duloxetine hydrochloride (hereafter referred to as duloxetine) was used at concentrations of 5, 10, and 20 μM (Fig. 1g–i). Treatment with duloxetine at 10 μM significantly increased the number of oligodendrocytes as compared to the MTZ-treated and recovery groups (Fig. 1j), and duloxetine significantly reduced the number of vacuolated cells compared to the control group (Fig. 1h,k). These results suggest that tacrine and duloxetine enhance the regeneration of oligodendrocytes in the spinal cord of zebrafish following demyelination.

**Figure 2:**
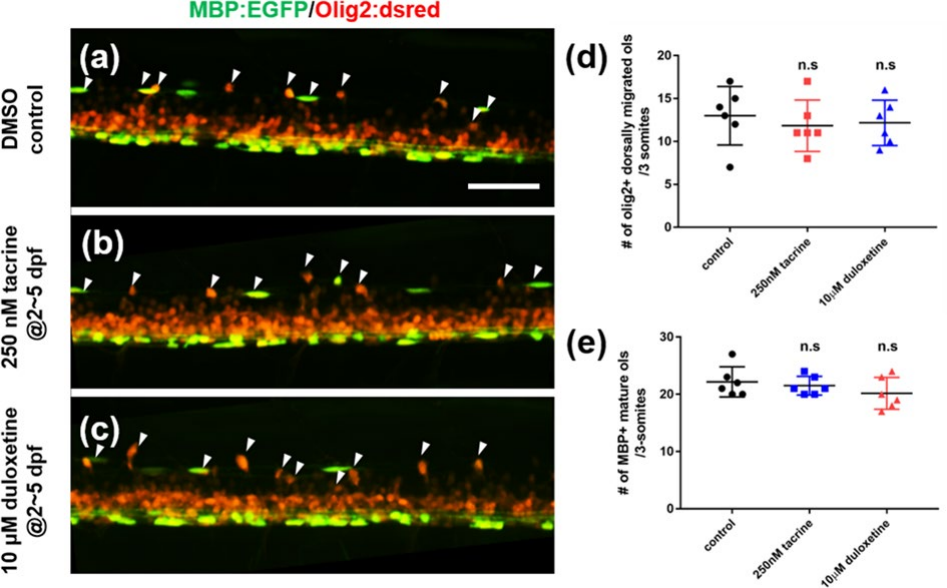
Tacrine and duloxetine show no effect on oligodendrocyte differentiation during development. All images are lateral views of the spinal cord of *Tg(mbpa:egfp;olig2:dsred)*, anterior to the left and dorsal to the top. Larvae were exposed to 0.2% DMSO (**a**), 250 nM tacrine (**b**), and 10 μM duloxetine (**c**) during dpf 2 to 5. Arrowheads indicate olig2 + dorsally migrated oligodendrocytes. (**d**) Quantification of the number of olig2 + dorsally migrated oligodendrocytes per 3-somite area. Control: 22.16 ± 2.64, 250 nM tacrine: 21.5 ± 1.64, 10 μM duloxetine: 20.16 ± 2.79. (**e**) Quantification of the number of MBP + mature oligodendrocytes per 3-somite area. Control: 13 ± 3.41, 250 nM tacrine: 11.8 ± 2.99, 10 μM duloxetine: 12.17 ± 2.64. n = 6 per group; n.s., not significant. Scale bar, 50 μm.

Given that tacrine and duloxetine restore the oligodendrocyte cell population after cell ablation, we assessed whether these two L1 mimetic compounds enhanced oligodendrocyte differentiation during the developmental process. To this end, we treated zebrafish larvae with each compound during oligodendrocyte differentiation. In the normal state, tacrine and duloxetine did not affect the differentiation of oligodendrocytes (Fig. 2a–c). The number of olig2 + dorsally migrated oligodendrocytes and mature MBP + oligodendrocytes was not different in either tacrine- or duloxetine-treated larvae compared to control larvae (Fig. 2d,e). However, unlike the normal state, L1 mimetic compounds, especially tacrine, increase cell proliferation in the spinal cord after oligodendrocyte ablation (Supplementary Fig. 3). These results indicate that tacrine and duloxetine effectively restore the oligodendrocyte population during demyelination and have no effect on normal development.

**Figure 3:**
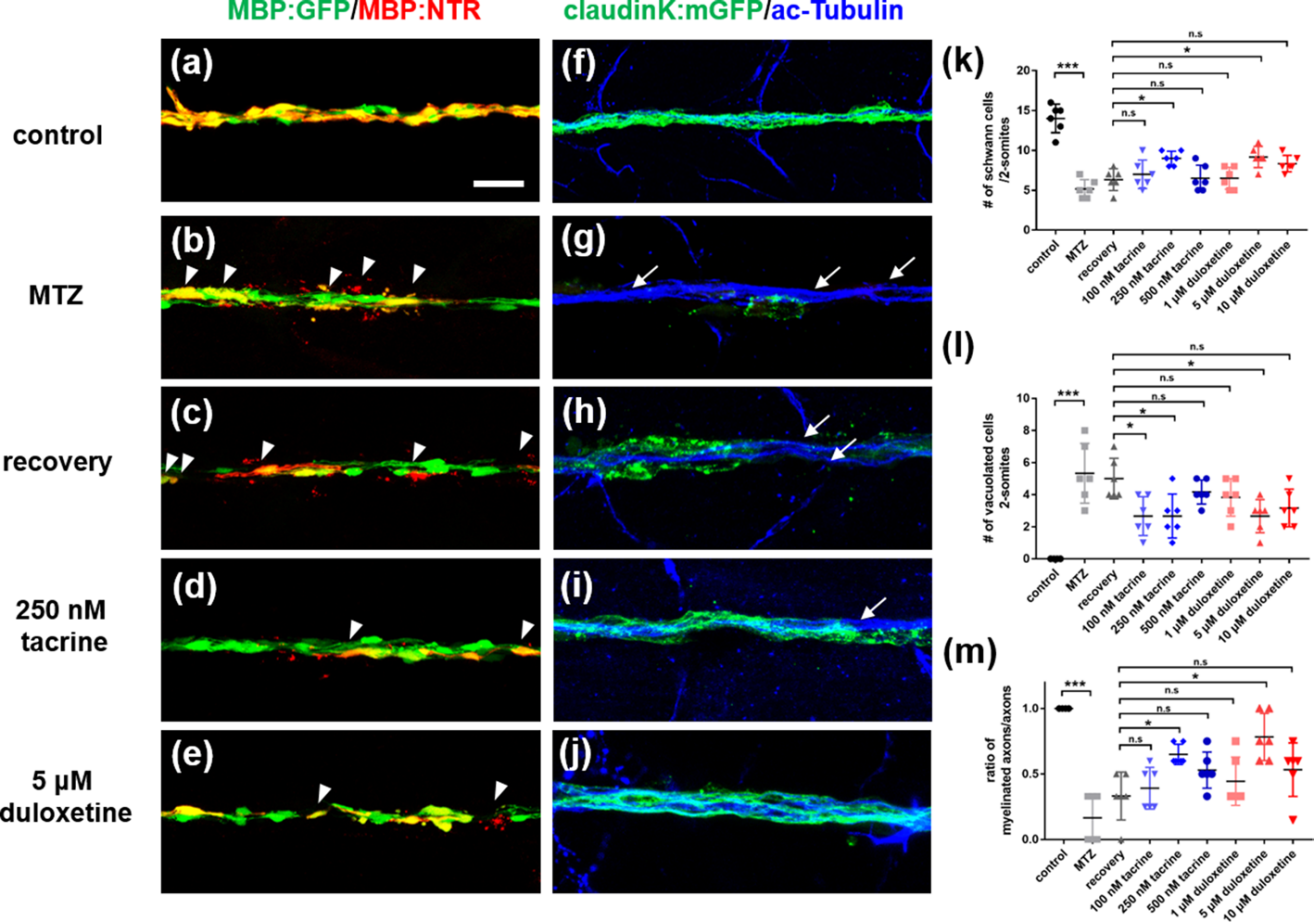
Tacrine and duloxetine enhance regeneration of Schwann cells in posterior lateral line axons after Schwann cell ablation. All images are lateral views of the posterior lateral line of (**a–e**) *Tg(mbpa:gal4-vp16;uas:egfp;uas:NTR-mCherry),* (**f–j**) *Tg(claudinK:gal4-vp16;uas:megfp;uas:NTR-mCherry)* larvae at 8 days post-fertilization (dpf). Anterior to the left and dorsal to the top. (**a–e**) GFP fluorescence indicates MBP-positive Schwann cells and red fluorescence indicates NTR + Schwann cells or vacuolated Schwann cells. Arrowheads indicate vacuolated cells. (**f–j**) GFP fluorescence indicates ClaudinK-positive myelin sheaths and blue fluorescence indicates acetylated tubulin-positive PLL axons. Arrows indicate demyelinated axons without GFP + myelin sheaths. (**k**) Quantification of the number of Schwann cells per 2-somite area. Control: 14 ± 1.79, MTZ: 5.16 ± 1.17, recovery: 6.33 ± 0.37, 100 nM tacrine: 7 ± 1.79, 250 nM tacrine: 9 ± 0.89, 500 nM tacrine: 6.5 ± 1.64, 1 μM duloxetine: 6.5 ± 1.33, 5 μM duloxetine: 9.17 ± 0.32, 10 μM duloxetine: 8.33 ± 1.03. (**l**) Quantification of the number of vacuolated cells per 2-somite area. Control: 0, MTZ: 5.33 ± 1.86, recovery: 5 ± 1.26, 100 nM tacrine: 2.67 ± 1.21, 250 nM tacrine: 2.67 ± 1.7, 500 nM tacrine: 4.17 ± 0.75, 1 μM duloxetine: 3.83 ± 1.17, 5 μM duloxetine: 2.27 ± 1.03, 10 μM duloxetine: 3.17 ± 1.17. (**m**) Ratio of the number of myelinated axons is indicated per total number of axons. Control: 1, MTZ: 0.165 ± 0.18, recovery: 0.33 ± 0.18, 100 nM tacrine: 0.39 ± 0.16, 250 nM tacrine: 0.65 ± 0.08, 500 nM tacrine: 0.53 ± 0.14, 1 μM duloxetine: 0.44 ± 0.18, 5 μM duloxetine: 0.78 ± 0.18, 10 μM duloxetine: 0.53 ± 0.2. n = 6 per group. The experiment was independently repeated three times. ***p < 0.001; **p < 0.01; *p < 0.05; n.s., not significant. Scale bar, 25 μm.

### Tacrine and duloxetine induce the regeneration of Schwann cells in the posterior lateral line after the ablation of Schwann cells

Next, we examined whether tacrine and duloxetine can promote the regeneration of Schwann cells in the peripheral nervous system after the ablation of Schwann cells. Schwann cells myelinate axons of the posterior lateral line (PLL) and the peripheral axons of motor neurons. GFP indicated MBP + Schwann cells in the PLL (Fig. 3a) and treatment of *Tg(mpba:gal4-vp16;uas:mgfp;uas:NTR-mCherry)* with MTZ-induced ablation of Schwann cells and increased vacuoles (Fig. 3b,k,l). After the washout of MTZ, L1 mimetic compounds, similar to their effect on the CNS, enhanced the regeneration of Schwann cells, compared with the recovery group (Fig. 3c–e,k,l). To visualize the PLL myelin sheaths with axons, we labeled *Tg(claudinK:gal4-vp16;uas:mgfp;uas:NTR-mCherry)* larvae with anti-acetylated tubulin antibody. Control larvae showed all axon fibers surrounded by ClaudinK^+^ myelin sheath (Fig. 3f). After MTZ treatment, these axons were completely demyelinated following Schwann cell ablation (Fig. 3g). To compare the degree of Schwann cell regeneration, we examined the ratio of GFP + myelinated axons to GFP- demyelinated axons (Fig. 3m). Tacrine and duloxetine significantly improved the cytological appearance of these axons compared with the MTZ-treated and recovery groups at concentrations of 250 nM and 5 μM, respectively (Fig. 3h–j,m). These findings indicate that both tacrine and duloxetine promote the regeneration of Schwann cells in the PLL axons after Schwann cell ablation.

### Tacrine and duloxetine induce remyelination in the central nervous system after demyelination

**Figure 4:**
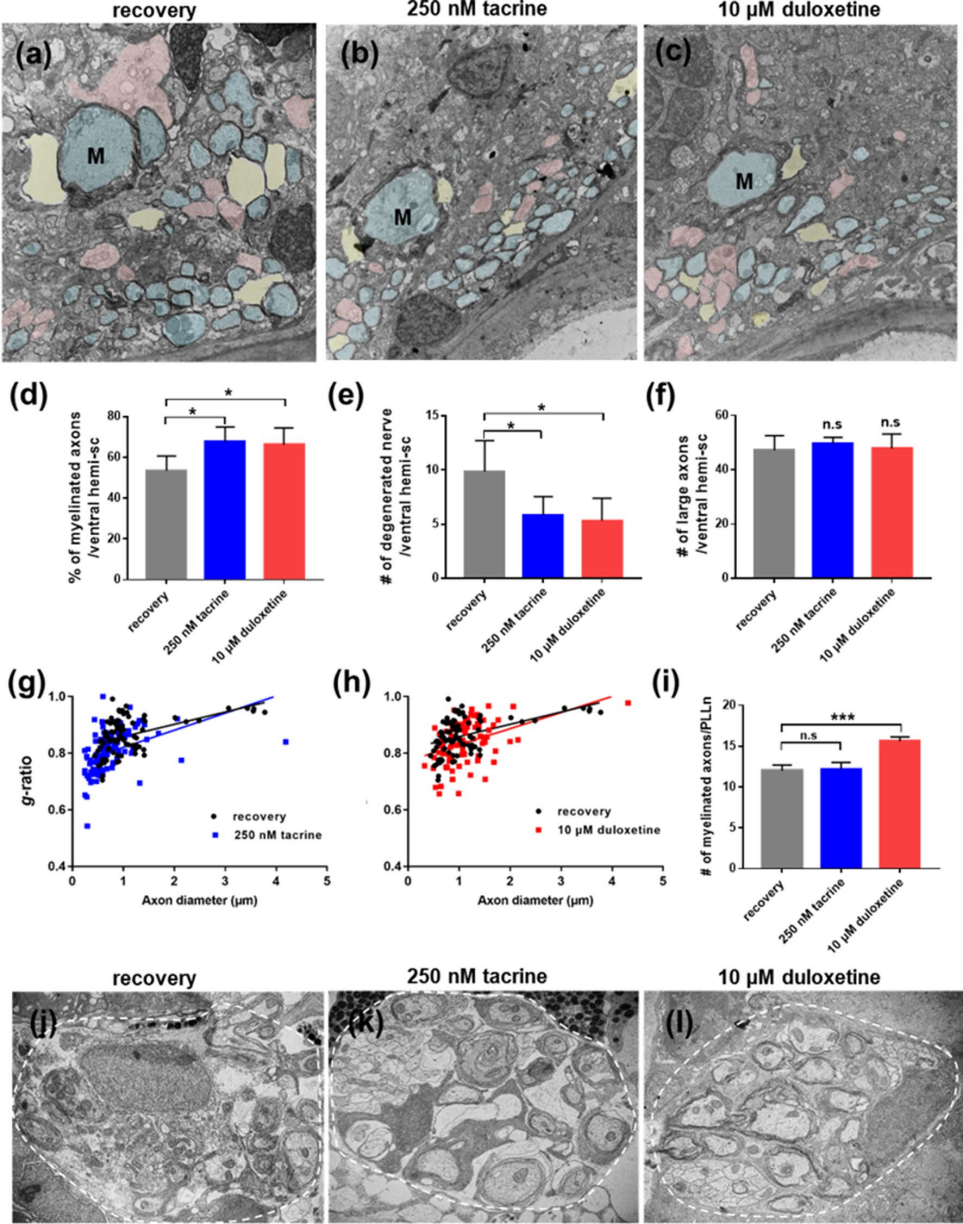
Tacrine and duloxetine promote remyelination after demyelination. Transmission electron microscopic images of transverse sections of the *Tg(mbp:gal4-vp16;uas:gfp;uas:NTR-mCherry)* larvae, with anterior to the left and dorsal to the top. (**a–c**) Representative images of ventral hemi-sections. M indicates Mauthner axon. Pseudo-colors were used to distinguish different degrees of myelination: blue indicates myelinated axons, pink indicates non-myelinated axons, and yellow indicates degenerated nerve structures. Scale bar, 2 μm. (**d**) Percentage of myelinated axons per ventral hemi-section. recovery: 53.4% ± 7.23, 250 nM tacrine: 69.2% ± 6.94, 10 μM duloxetine: 66.17% ± 8.23. (**e**) Quantification of the number of degenerated nerve structures per ventral hemi-section. Recovery: 9.86 ± 2.85; 250 nM tacrine: 5.83 ± 1.72; 10 μM duloxetine: 55.3 ± 2.07. (**f**) Quantification of the number of large caliber axons (> 1 μm) per ventral hemi-section. Recovery: 47.2 ± 5.36; 250 nM tacrine: 49.6 ± 2.30; 10 μM duloxetine: 47.8 ± 5.36. n = 5 for the recovery groups; n = 6 for the tacrine and duloxetine groups (**g, h)** Decreased g-ratio in myelinated axons in tacrine-treated larvae (blue line) and duloxetine-treated larvae (red line) compared to MTZ-treated larvae (black line); g-ratios: 0.86 ± 0.063; 250 nM tacrine: 0.81 ± 0.072, p < 0.001; 10 μM duloxetine: 0.4 ± 0.073, p < 0.05; n = 3. (**i**) Quantification of the number of myelinated axons per PLLn. Recovery: 12 ± 0.71, 250 nM tacrine: 12.2 ± 0.84, 10 μM duloxetine: 15.6 ± 0.55. (**j–l**) Representative sectioned images of PLL. Scale bar, 1 μm. ***p < 0.001; *p < 0.05; n.s., not significant.

To determine whether tacrine and duloxetine can induce remyelination in the demyelinated nervous system as well, transmission electron microscopy (TEM) was performed on transverse sections of the spinal cord and PLL (Fig. 4). Axons in the spinal cord and PLL of untreated control larvae were surrounded by a compact and thick myelin sheath (Supplementary Fig. 4a,c). Upon MTZ treatment, larvae showed robust demyelination compared to control larvae (Supplementary Fig. 4b,d). The percentage of myelinated axons per ventral hemi-spinal cord was decreased in MTZ-treated larvae and demyelination was accompanied by substantial degeneration of axons associated with myelin debris (Supplementary Fig. 4e–h). In contrast to the recovery group, tacrine- and duloxetine-treated larvae showed higher numbers of remyelinated axons (Fig. 4a–c). Tacrine and duloxetine treatment increased the percentage of myelinated axons and concomitantly decreased the number of degenerated axons (Fig. 4d,e). No significant differences were observed in the number of large axons between the different groups (Fig. 4f). To quantitatively evaluate myelin thickness, the g-ratio (the ratio of the inner diameter to the outer diameter of the myelin sheath) was determined. The tacrine- and duloxetine-treated groups exhibited a reduced g-ratio compared to the recovery group, indicating the promotion of remyelination (Fig. 4g,h). Next, we examined whether tacrine and duloxetine promoted remyelination in the peripheral nervous system after demyelination. In the PLL, TEM analysis showed that only the duloxetine-treated group displayed enhanced remyelination compared to the recovery group (Fig. 4i,j,l). However, unlike the CNS, tacrine showed little effect on remyelination in the PLL (Fig. 4i,k**)**. Together, these results demonstrate that tacrine and duloxetine enhance regeneration of myelinating glia and remyelination.

Demyelination induces the activation and migration of macrophages/microglia resulting in the rapid removal of immune cells which is necessary for the remyelination step^19^. In order to verify whether L1 mimetic compounds enhance remyelination by modulating the immune response induced by oligodendrocyte ablation, we examined *Tg(mpeg:gfp;mbpa:gal4-vp16;uas:NTR-mCherry)* larvae, which express GFP in the microglia, and found that *mpeg*:GFP^+^ microglia were recruited in the spinal cord after the ablation of oligodendrocytes (Supplementary Fig. 5a,d). Interestingly, duloxetine-treated larvae exhibited a significant reduction in *mpeg*:GFP^+^ cells in the spinal cord (Supplementary Fig. 5c,d), but tacrine treatment had no effect on the immune response (Supplementary Fig. 5b,d). These results indicate that duloxetine, but not tacrine, improves the remyelination process by reducing excessive neuroinflammation.

**Figure 5:**
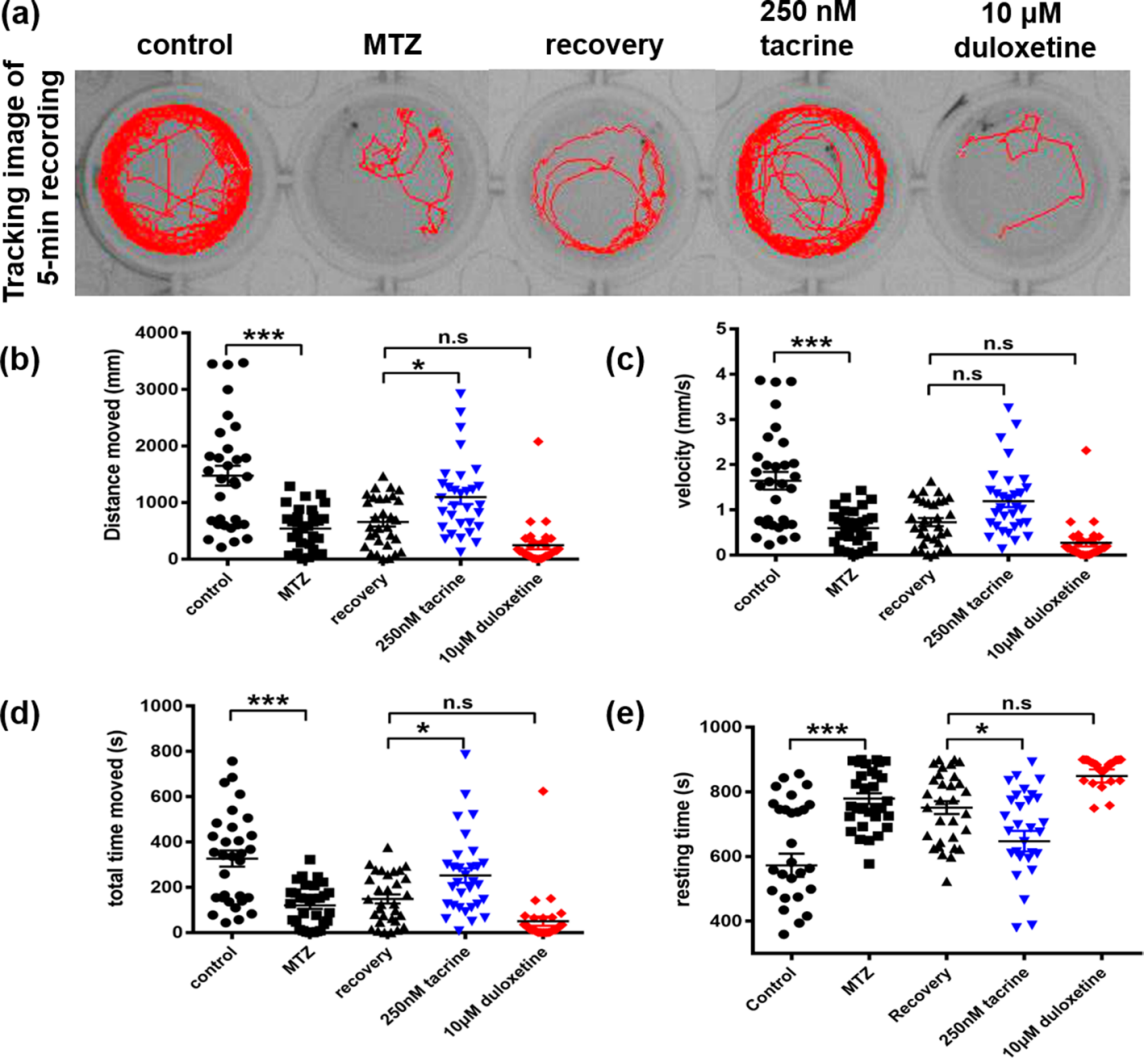
Tacrine, but not duloxetine, promotes restoration of locomotor activity. (**a**) Representative tracking image of a 5-min recording at 8 dpf. Quantification of (**b**) total distance moved (control: 1476.41 ± 975.90, MTZ: 539.10 ± 369.30, recovery: 655.05 ± 441.90, 250 nM tacrine: 1093.44 ± 670.50, 10 μM duloxetine: 243.87 ± 383.45), (**c**) velocity (control: 1.64 ± 1.09, MTZ: 0.60 ± 0.41, recovery: 0.73 ± 0.49, 250 nM tacrine: 1.19 ± 0.75, 10 μM duloxetine: 0.27 ± 0.42), (**d**) total movement time (control: 327.08 ± 200.34, MTZ: 120.81 ± 90.72, recovery: 148.80 ± 109.57, 250 nM tacrine: 252.45 ± 176.18, 10 μM duloxetine: 50.55 ± 113.43), and (**e**) total resting time (control: 572.91 ± 200.34, MTZ: 779.18 ± 90.72, recovery: 751.19 ± 109.57, 250 nM tacrine: 647.54 ± 176.18, 10 μM duloxetine: 549.44 ± 113.43) during 15-min recordings. The experiment was independently repeated three times (n = 31 per group). Error bars indicate mean ± standard deviation. ***p < 0.001; **p < 0.01; *p < 0.05; n.s., not significant.

### Tacrine restores locomotor activity after demyelination

MTZ-induced demyelination has been shown to lead to locomotor deficits in zebrafish larvae^20^. We therefore investigated whether the observed remyelination was sufficient to permit functional improvements. To this end, we measured parameters of locomotor activity, that is, distance moved (mm), velocity (mm/s), movement time (s), and resting time (s) over a time period of 20 min. These parameters were robustly reduced in MTZ-treated *tg (mbpa:gal4-vp16;uas:NTR-mCherry*) larvae compared to control larvae (Fig. 5). Since MTZ-treated wild type larvae showed normal locomotor activity (Supplementary Fig. 6), we conclude that these defects in motor activity were due to demyelination. In addition, both the MTZ-treated and recovery groups showed similar locomotor activity, despite our finding that the recovery group showed a tendency to partially restore oligodendrocytes (Fig. 1c). These observations suggest that due to the short recovery time, functional recovery was not achieved. In contrast, treatment with 250 nM tacrine significantly increased locomotor activity compared to the recovery group (Fig. 5). Interestingly, locomotor activity was decreased in the duloxetine-treated group, in comparison with not only the recovery group but also the MTZ-treated transgenic zebrafish, which may be explained by the sedative effects of this compound. To investigate its sedative effects, we tested whether duloxetine treatment also affects the behavior of the wild type. Duloxetine-treated wild type zebrafish showed very low locomotor activity (Supplementary Fig. 6). In addition, the a*ctin* cytoskeleton of the skeletal muscles was not affected by duloxetine (Supplementary Fig. 7), indicating no noticeable effect on the viability of other cell types. We next tested whether functional recovery was achieved after washout of duloxetine. Locomotor behavior was examined for another 2 days after washout. The sedative effects of duloxetine weakened over time, returning to the level of the recovery group within 1 day (Supplementary Fig. 8). Taken together, these observations provide evidence that the decrease in locomotor activity observed with duloxetine was due to a sedative effect. Therefore, tacrine, in contrast to duloxetine, effectively promotes remyelination following demyelination and thereby boosts functional recovery*.*

## Discussion

In this study, we used a robust and reliable transgenic zebrafish demyelination model to evaluate the effects of the L1 mimetic agonist compounds tacrine and duloxetine, which have previously been shown to enhance regeneration in mammalian cell cultures and the spinal cord^14–16^. Here, we found that these compounds displayed beneficial effects in both the peripheral and central nervous system of the demyelinated zebrafish, as evidenced by the increased survival and restoration of oligodendrocytes, protection of axons, improved myelin compaction and thickness, and, importantly, recovery of locomotive functions as indicated by several parameters of locomotor activity. These results show that our selected agonistic L1 mimetic compounds improved recovery after demyelination and demonstrate the feasibility and significance of the zebrafish demyelination model for screening and validating drug candidates for the improvement of remyelination.

It should be emphasized that the two evaluated L1 mimetics are known to exert other functions in addition to mimicking L1. Several acetylcholinesterase inhibitors including tacrine, rivastigmine, and donepezil are used in the treatment of individuals with moderate Alzheimer’s disease by inhibiting the breakdown of acetylcholine^21^. However, tacrine has been removed from the list of FDA-approved drugs since adverse effects were observed at the prescribed concentrations^22^. Oligodendrocyte lineage cells express several nicotinic acetylcholine receptors, depending on their developmental stage^23^. Cholinergic signaling is important for oligodendrocyte development and myelination under normal physiological conditions and in disease. Thus, acetylcholinesterase inhibitors were reported to induce myelin gene expression, improve myelin integrity, and reduce inflammation^24^. Being an acetylcholinesterase inhibitor, tacrine may therefore not only act as an L1 agonist but also affect the differentiation of oligodendrocytes, which may well have played a role in the effects on remyelination observed here. Recent research has shown that donepezil, an acetylcholinesterase inhibitor, promotes the differentiation of oligodendrocyte precursor cells to oligodendrocytes in in vitro cell culture systems, while tacrine does not^25^. However, the current study shows that tacrine has the capacity to restore oligodendrocyte cell populations in a pathological state, such as demyelination. Therefore, although tacrine has been taken off the market due to adverse effects in relation to Alzheimer’s disease, and despite the fact that findings in zebrafish are not directly translatable to a human context^26^, we would still like to suggest that lower tacrine concentrations may be used in humans since we observed regenerative effects of this drug in the nanomolar range in our experiments.

Here we show that in zebrafish, similar to tacrine, treatment with duloxetine protects oligodendrocytes from degeneration while boosting their regeneration, thereby enhancing remyelination. Duloxetine is known to affect neuroinflammation by regulating cytokine responses^27^, namely by inhibiting the release of pro-inflammatory cytokines while stimulating the production of anti-inflammatory cytokines. Also, in a mouse model of neuropathic pain, duloxetine inhibits immune responses via downregulation of the tumor necrosis factor-α (TNF-α)/NF-κB signaling pathway^28^. Since multiple pro-inflammatory cytokines including TNF-α are thought to contribute to inflammation and demyelination in MS^29^, it is possible that in the current study, duloxetine improved remyelination by reducing inflammation and thus creating a protective and pro-regenerative environment. Contrary to tacrine-treated larvae, duloxetine-treated larvae did not show improved functional locomotive recovery. Although duloxetine has been reported to induce hyperactivity in several animal models, other studies have observed deviating effects of this compound^30–32^. Duloxetine treatment may affect the behavior of zebrafish larvae via 5-HT4 and α_2_-adrenergic receptors in the central and peripheral nervous system and has been reported to cause a decrease in swimming speed and an increase in resting time, which is consistent with our results^33^. Since it has been shown that the effect of duloxetine on locomotor activity varies depending on concentration and treatment time^34^, further studies are required to elucidate whether this L1 mimetic may improve functional recovery following demyelination at other doses.

Altogether, the present study shows that tacrine and duloxetine enhance remyelination in a zebrafish demyelination model. These beneficial effects are likely due to a combination of L1′s ability to affect myelination and the compounds’ indirect effects on the animal’s immune system. The fact that L1 promotes myelination in mammalian cell cultures in the absence of immune system cells^13^ is indicative of its direct influence on myelination. The overall effects of these two L1 mimetics on enhancing remyelination raises the hope that tacrine and duloxetine may be repurposed for the treatment of demyelinating diseases.

## Materials and methods

### Zebrafish lines

*Tg(mbpa:gal4-vp16)*, *Tg(uas:NTR-mCherry)*^11^, *Tg(claudinK:gal4-vp16;uas:mgfp)*^35^, *Tg(uas:egfp)*^36^ and *Tg(mpeg:gfp)*^37^ lines of either sex were used.

### Treatment with metronidazole and L1 mimetic compounds

Metronidazole (MTZ, Cat #M1547, Sigma-Aldrich, St. Louis, MO, USA) was dissolved in embryo medium containing 0.2% DMSO to yield a final concentration of 10 mM as described previously^11^. Tacrine hydrochloride (Cat #1648–40-8, Tocris, Illkirch, France) and (S)-duloxetine hydrochloride (Cat #136,434–34-9, Tocris) were dissolved in distilled water to yield a 10-mM stock solution. For cell ablation, NTR-transgenic larvae were exposed to 10 mM MTZ for 2 days from 5 to 7 dpf. To test remyelination, MTZ was subsequently washed out using embryo medium, and larvae were incubated in embryo medium containing tacrine, duloxetine, or vehicle (recovery group) for 1 day (Supplementary Fig. 1) prior to analyses.

### Immunohistochemistry and imaging

For whole-mount immunohistochemistry, larvae were immersed overnight in 4% formaldehyde at 4 °C, washed three times at room temperature in phosphate-buffered saline (PBS)/0.02% Triton X-100, pH 7.3, incubated in 2% BSA with 5% sheep serum (Cat #013–000-12, Jackson Immuno, West Grove, PA, USA) for 2 h at room temperature, and then incubated overnight with primary antibodies at 4 °C. For labeling axons, mouse anti-acetylated tubulin (1:1000, Cat #T6793, Sigma) and Alexa 647-conjugated secondary antibodies (1:500, Cat #A-21235, Molecular Probes, Thermo Fisher Scientific, Waltham, MA, USA) were used.

All fluorescent images were taken in the 2-somite area above the end of the yolk extension using an A1Si laser-scanning confocal microscope (Nikon, Tokyo, Japan). Confocal images (1-μm z-stacking) were processed using NIS-Elements AR Analysis 4.30 software (Nikon).

### Transmission electron microscopy

Tissues were prepared using standard procedures as previously described^38^. Zebrafish larvae were anesthetized with tricaine (Cat #A5040, Sigma) and fixed in 10% formaldehyde/2.5% glutaraldehyde/0.1 M phosphate buffer, with a pH of 7.4, at 4 °C overnight. Then, larvae were post-fixed in 1% osmium tetroxide, sequentially dehydrated, and embedded in eponate-12 resin (Cat #18,006, Ted Pella). Sections of 1-μm thickness were obtained using a Reichert-Jung Ultracut E Ultramicrotome (Leica Microsystems, Wetzlar, Germany) and stained with toluidine blue. Images were taken with a Zeiss Axio Observer microscope (Carl Zeiss Microscopy, Jena, Germany). Sections of 60-nm thickness were collected on formvar-coated slot grids, stained with uranyl acetate/lead citrate, and imaged on a H-7500 transmission electron microscope (80 kV, Hitachi, Tokyo, Japan).

### Behavior

All behavior tests were performed between 1 and 4 pm in light conditions. At 8 dpf, larvae from each group were individually transferred onto 48-well plates, with each well containing 500 μl of embryo medium. For the behavioral analyses, larvae were acclimated for 1 h before their swimming performance was recorded. Then, locomotion was recorded for 15 min under light conditions and analyzed using the EthoVision XT 12 system (Noldus, Wageningen, Netherlands). The total distance moved was recorded in mm, velocity in mm/s, and total time moved in seconds, each over the course of 15 min. Each test was independently repeated three times.

### Statistics

Statistical analyses were conducted using GraphPad Prism 7.0c software (GraphPad Software, San Diego, CA, USA). A one-way analysis of variance (ANOVA) followed by Tukey’s test for multiple comparisons was used to analyze differences between the groups. Comparisons of g-ratios were performed using linear regression analysis. Data are expressed as the mean ± standard deviation and *p* < 0.05 was considered to indicate a significant difference. Statistical significances are indicated as follows: *p < 0.05; **p < 0.01; and n.s. (not significant).

### Ethics approval

All experimental procedures were approved by the Korea University Institutional Animal Care and Use Committee and carried out in accordance with the animal experimental guidelines of the Korea National Veterinary Research and Quarantine Service. The study was carried out in compliance with the ARRIVE guidelines.

## Supplementary Information

Supplementary Figures.

## Data Availability

Data supporting the findings of this study are available in the article and the Supplementary Information Files, or from the corresponding authors on reasonable request.
