# Supplementary material for: Small compounds mimicking the adhesion molecule L1 improve recovery in a zebrafish demyelination model
Source: Sci Rep. 2021 Mar 15;11:5878. doi: 10.1038/s41598-021-85412-1 (PMC7960995; doi:10.1038/s41598-021-85412-1)
Supplement: Supplementary file 1 — Supplementary Figures. [file 41598_2021_85412_MOESM1_ESM.docx]

**Small compounds mimicking the adhesion molecule L1 improve recovery**

**in a zebrafish demyelination model**

Suhyun Kim^1^, Dong-Won Lee^1^, Melitta Schachner^2, 3, *^ and Hae-Chul Park^1, *^

^1^Department of Biomedical Sciences, College of Medicine, Korea University, Ansan 15335, Republic of Korea; ^2^Keck Center for Collaborative Neuroscience and Department of Cell Biology and Neuroscience, Rutgers University, Piscataway, NJ 08554, USA; ^3^Center for Neuroscience, Shantou University Medical College, Shantou, Guangdong 515041, China

***Corresponding authors**

Hae-Chul Park: Email: [hcpark67@korea.ac.kr](mailto:hcpark67@korea.ac.kr), Telephone: +82-31-412-6713

Melitta Schachner: Email: [schachner@dls.rutgers.edu](mailto:schachner@dls.rutgers.edu), Telephone: +1-848-445-1780


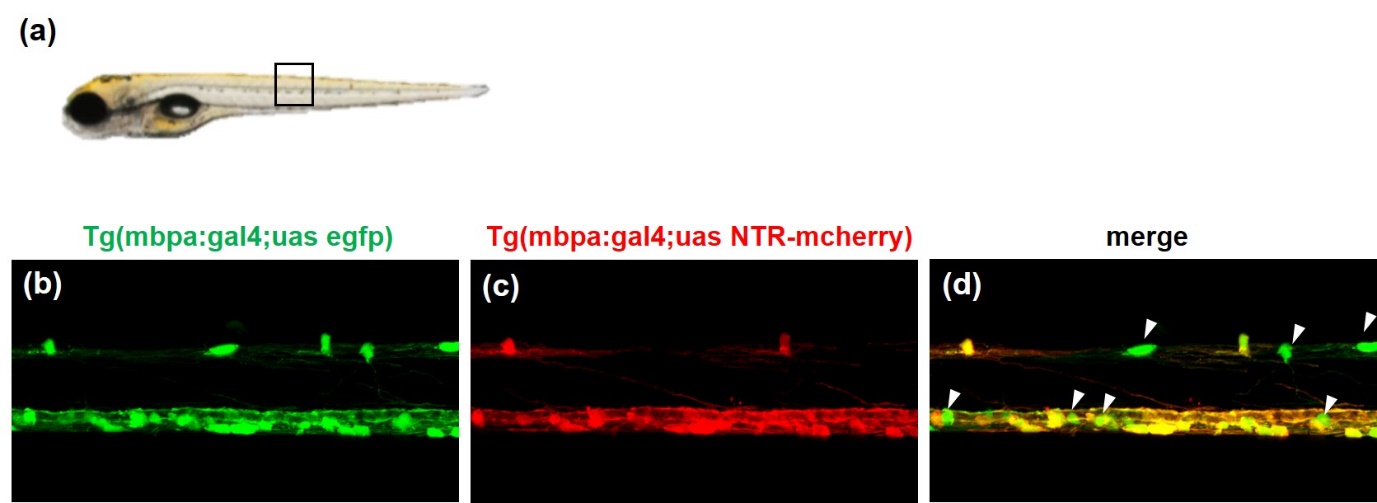


**Supplementary Figure 1.** **Expression of *Tg(mbpa:gal4-vp16;uas:NTR-mCherry*)**

(**a**) All fluorescent images were taken in the 2-somite area above the end of the yolk extension. (**b–d**) Lateral views of the trunk of *Tg(mbp:gal4-vp16;uas:NTR-mCherry)* larvae; anterior to the left and dorsal to the top. To examine efficiency of NTR expression, *Tg(mbp:gal4-vp16;uas:NTR-mCherry)* larvae were crossed with *Tg(uas:egfp).* In our transgenic system, NTR was expressed in almost 60% of MBP^+^ cells.


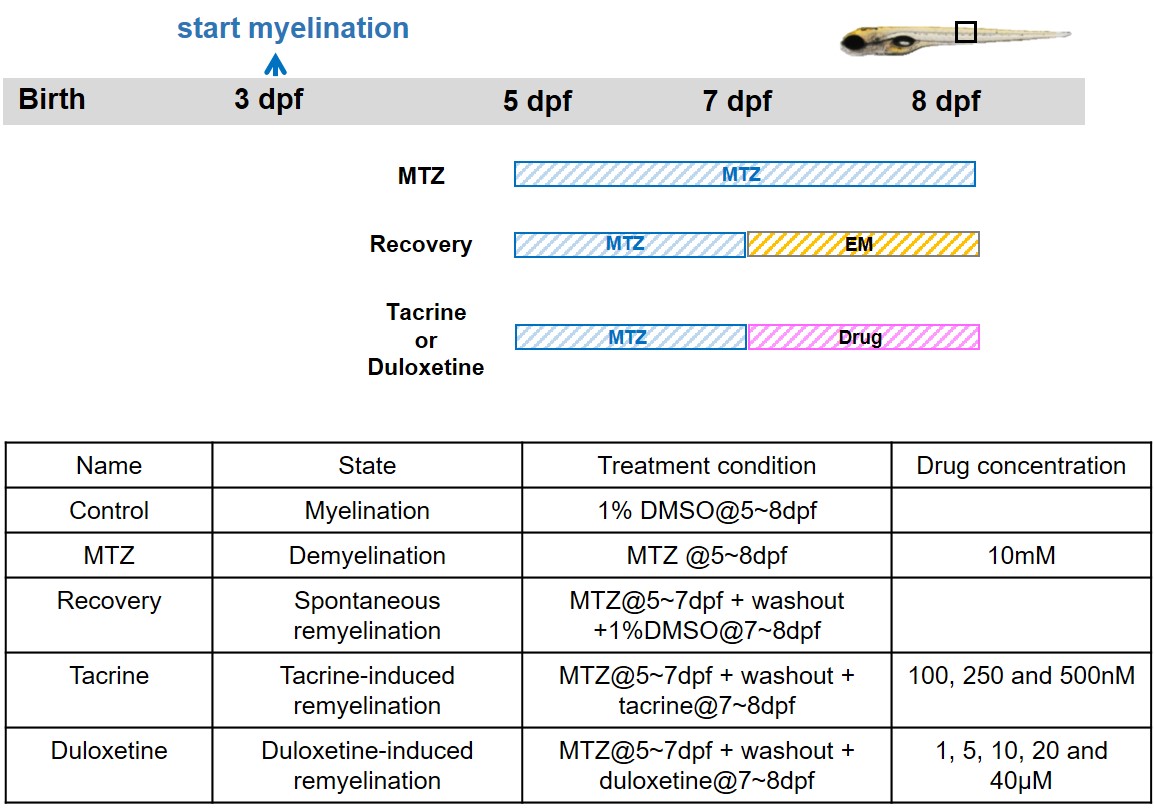


**Supplementary Figure 2. Summary scheme of the experimental conditions**


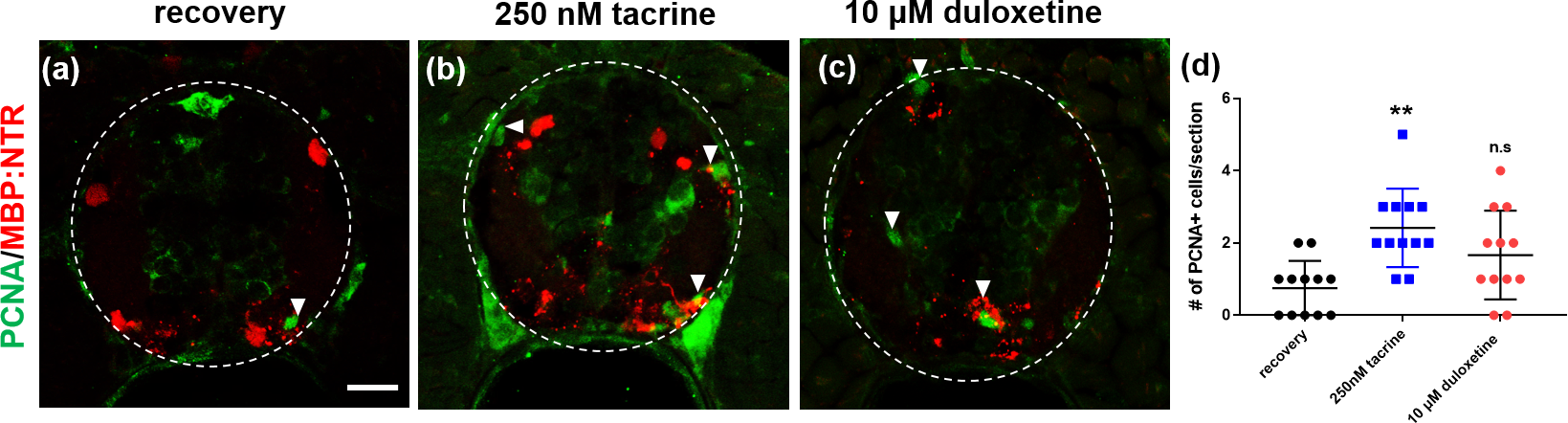


**Supplementary Figure 3. Tacrine and duloxetine induce proliferation after cell ablation**

(**a–c**) All images are transverse sections of the spinal cord of *Tg(mbpa:gal4-vp16;uas:NTR-mCherry)* larvae (top indicates dorsal). IHC labeling of recovery (**a**), tacrine (**b**), and duloxetine (**c**)-treated larvae at 8 dpf with anti-anti-PCNA antibody (green). Arrowheads indicate PCNA^+^ cells in the white matter. Scale bar, 1 μm. (**d**) Quantification of the number of PCNA^+^ cells per section. Recovery: 0.75±0.75; 250 nM tacrine: 2.41±1.08; 10 μM duloxetine: 1.66±1.23. n=12 for each group.

**
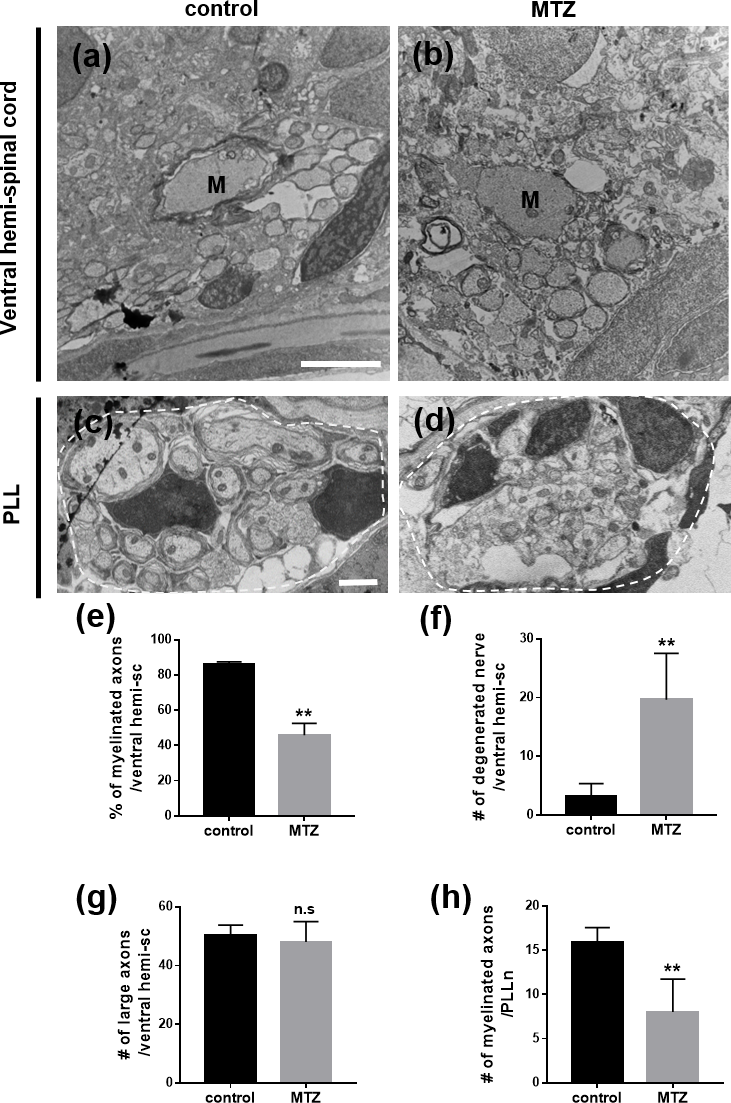
**

**Supplementary Figure 4. Ablation of MBP^+^ cells results in extensive demyelination**

Transmission electron microscopic images of transverse sections of the of the spinal cord of *Tg(mbp:gal4-vp16;uas:gfp;uas:NTR-mCherry)* larvae, with anterior to the left and dorsal to the top. (**a–b**) Representative images of ventral hemi-spinal cords. M indicates Mauthner axon. Scale bar, 2 μm. (**c–d**) Representative sectioned images of PLL. Scale bar, 1 μm. White dashed circle indicates the area of PLL. (**e**) Percentage of myelinated axons per ventral hemi-section. Control: 86%±1.58, MTZ: 45.8%±6.76. (**f**) Quantification of the number of degenerated nerve structures per ventral hemi-section. Control: 3.2±2.17; MTZ: 19.6±7.92. (**g**) Quantification of the number of large caliber axons (>1 μm) per ventral hemi-section. Control: 50.4±3.36; MTZ: 48±6.96. (**h**) Quantification of the number of myelinated axons per PLLn. Control: 15.83±1.72; MTZ: 8±3.74. n=5 for each group.

**
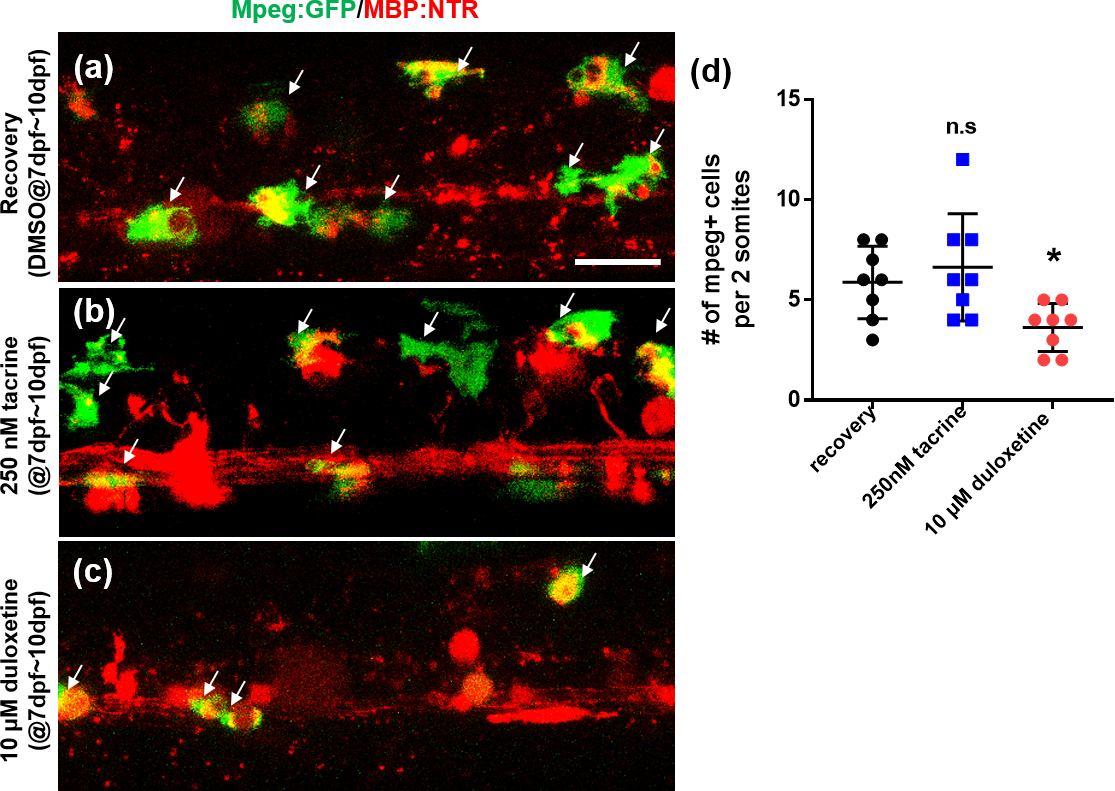
**

**Supplementary Figure 5. Duloxetine inhibits excessive neuroinflammation in demyelinated spinal cord**

**(a–c**) All images are lateral views of the spinal cord of *Tg(mpeg:gfp;mbpa:gal4-vp16;uas:NTR-mCherry)* larvae at 10 days post-fertilization (dpf), anterior to the left and dorsal to the top. Arrows indicate mpeg+ microglia and macrophages. Scale bar, 25 μm. (**d**) Quantification of the number of mpeg^+^ cells per 2-somite area. Recovery: 5.87±1.81; 250 nM tacrine: 6.63±2.67; 10 μM duloxetine: 3.62±1.19. n=8 for each group.

**
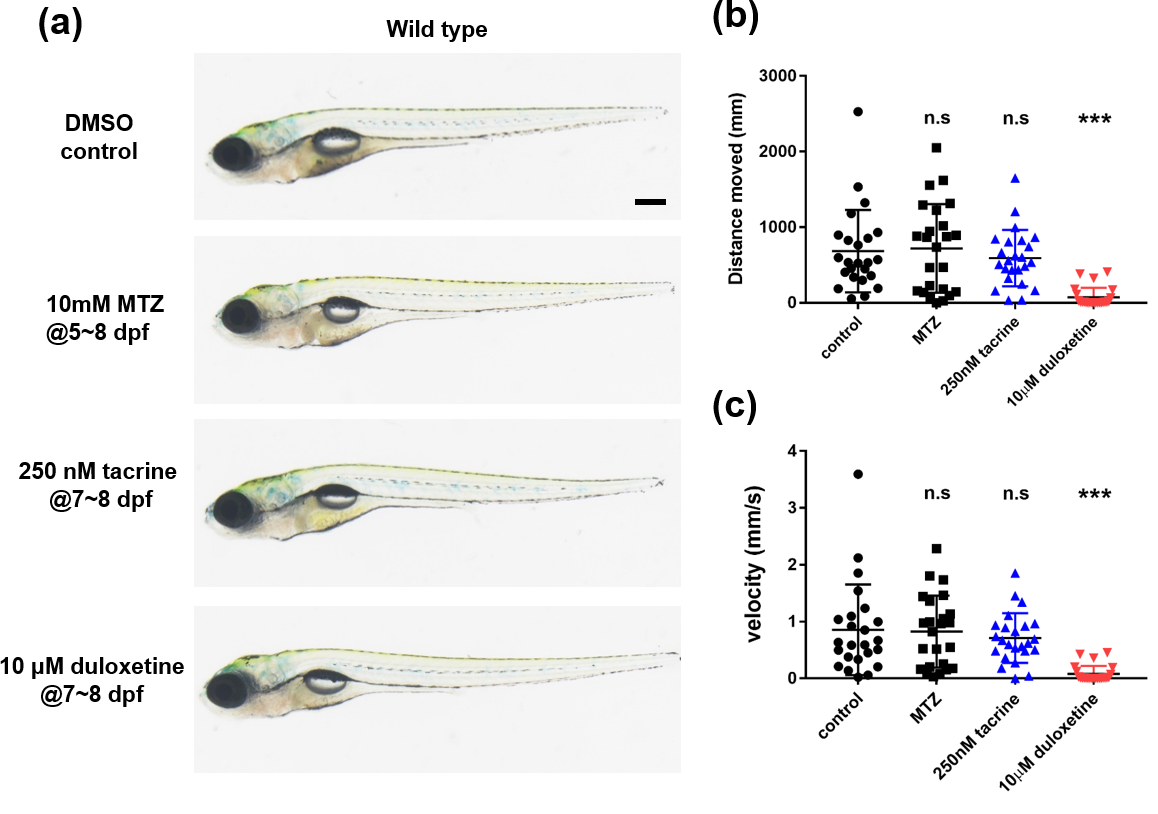
**

**Supplementary Figure 6. Wild type animals treated with MTZ or L1 mimetic compounds show normal morphological phenotype and locomotor activity**

(**a**) All images are lateral views of the trunk of 8-dpf wild type larvae; anterior to the left and dorsal to the top. (**b**) Total distance moved (control: 684.233±545.34; MTZ: 719.43±586.17; 250 nM tacrine: 591.81±373.10; 10 μM duloxetine: 72.77±127.05), (**c**) velocity (control: 0.86±0.79; MTZ: 0.82±0.63; 250 nM tacrine: 0.71±0.44; 10 μM duloxetine: 0.08±0.14), n = 24 per group. ***p < 0.001; n.s., not significant.


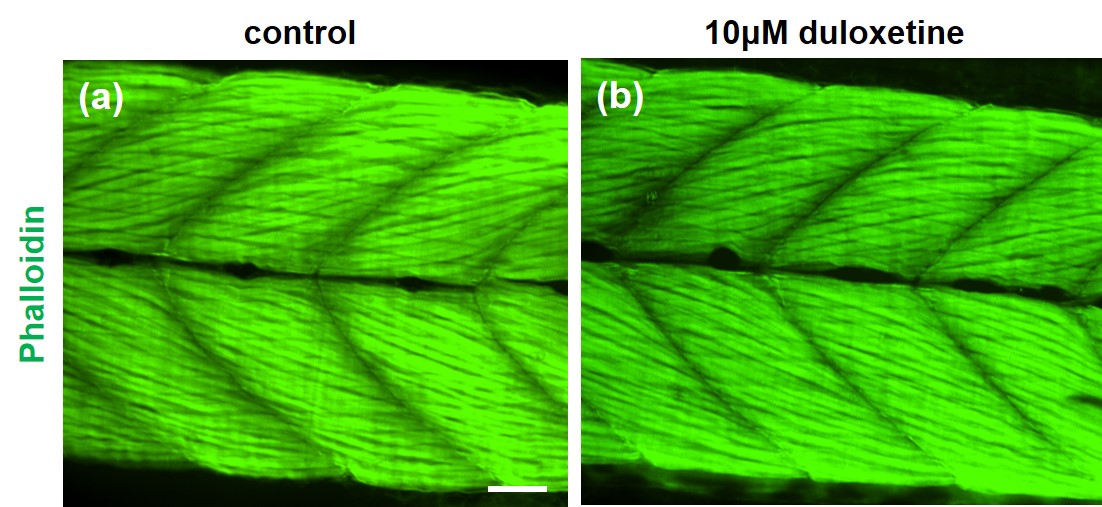


**Supplementary Figure 7. Duloxetine treatment does not affect muscle structure**

All images are lateral views of the trunk of *Tg(mbp:gal4-vp16;uas:NTR-mCherry)* larvae; anterior to the left and dorsal to the top. For visualization of actin cytoskeleton in muscles, larvae were labeled with Alexa Fluor 488 phalloidin. No difference was observed between muscles of larvae treated with duloxetine (**b**) and those of untreated control larvae (**a**).


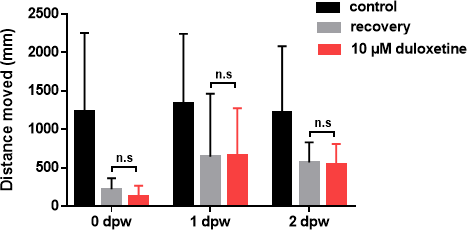


**Supplementary Figure 8. Washout of duloxetine eliminates the sedative effect**

Quantification of total distance moved (mm) over the course of 15 min. After 24 h of exposure in an embryonic medium containing 1% DMSO (recovery group) or 10 μM duloxetine, the solutions were washed out and refreshed with embryonic medium. The behavior of larvae was monitored every 24 h from 0 dpw (days-post-washout) to 2 dpw. Within 2 days of the duloxetine removal, the sedative effect weakened, and the locomotor behavior was restored to about 50% of control level. However, exposure to duloxetine did not affect locomotor activity as it was not increased from that of the recovery group. n = 24 for control, n=10 for recovery and duloxetine groups. Data represent the mean ± SEM.
